# Improved Generation of Induced Pluripotent Stem Cells From Hair Derived Keratinocytes – A Tool to Study Neurodevelopmental Disorders as ADHD

**DOI:** 10.3389/fncel.2018.00321

**Published:** 2018-09-25

**Authors:** Silvano Re, Asli Aybike Dogan, Dorit Ben-Shachar, Gregor Berger, Anna Maria Werling, Susanne Walitza, Edna Grünblatt

**Affiliations:** ^1^Department of Child and Adolescent Psychiatry and Psychotherapy, University Hospital of Psychiatry Zurich, University of Zurich, Zurich, Switzerland; ^2^Laboratory of Psychobiology, Department of Psychiatry, Rambam Health Care Campus, B. Rappaport Faculty of Medicine, Rappaport Family Institute for Research in Medical Sciences, Technion-Israel Institute of Technology, Haifa, Israel; ^3^Neuroscience Center Zurich, University of Zurich, ETH Zurich, Zurich, Switzerland; ^4^Zurich Center for Integrative Human Physiology, University of Zurich, Zurich, Switzerland

**Keywords:** iPSCs, keratinocyte, hair follicle, reprogramming, ADHD, neurodevelopment, disease modeling, stem cell

## Abstract

In the last decade, there is an increasing application of induced pluripotent stem cells (iPSCs) for disease modeling. The iPSC technology enables the study of patient-specific neuronal cell lines *in vitro* to evaluate dysfunction at the cellular level and identify the responsible genetic factors. This approach might be particularly valuable for filling the gap of knowledge at the cellular and molecular levels underlying the pathophysiology of various neurodevelopmental and/or psychiatric disorders, such as attention-deficit hyperactivity disorder (ADHD). However, the invasiveness of skin biopsy or blood withdrawal might represent a major impediment in such protected population. Using hair derived keratinocytes as starting somatic cells circumvents this problem as sample collections can be performed non-invasively. Here we describe an improved, convenient, standardized and effective method to culture and reprogram hair derived keratinocytes from three healthy controls and one ADHD patient into iPSCs, which in turn will be used to generate differentiated neuronal cells. All the cell types were maintained in highly defined, serum-free conditions and showed expression of the respective key marker genes, assessed by both immunocytochemistry and qRT-PCR. The described *in vitro* personalized neuronal model has its advantage in modeling neurodevelopmental trajectories since it can recapitulate key processes of brain development at the cellular and molecular level and is intended to be used as for example studying ADHD etiopathology.

## Introduction

The molecular and cellular mechanisms involved in neurodevelopmental disorders like attention-deficit hyperactivity disorder (ADHD) are often poorly understood, mainly due to inaccessibility to brain tissue and limitations of the available model systems.

As it is the case in other neurodevelopmental disorders, investigating the etiopathology of ADHD at the molecular level is limited by a multitude of factors. Conventional *in vitro* and *in vivo* model systems lack the patient-specific genetic background and the complexity of the human brain. With over 70% heritability, ADHD demonstrates high genetic load (Demontis et al., 2017); nevertheless, it is postulated to have a polygenetic character as no high penetrance mutation has yet been associated (Neale et al., 2010). Genetically modified animal models have recreated some of the prevailing symptoms of ADHD, but none of these models managed to reflect the disorder faithfully enough to lead to significant clinical implication (Giros et al., 1996; Carvalho et al., 2016). The same applies to peripheral biological samples, which fail to reveal enough information about the dysfunction in the central nervous system, while on the other hand, post-mortem brain tissues do not give sufficient insights into the abnormal process occurring during development and are rarely available for research purposes. Therefore, there is an urgent need to better understand the pathophysiology of the disorder in order to develop more effective, practical and individually tailored pharmaceutical therapies and to discover new predictive biomarkers.

An interesting candidate for *in vitro* modeling of neurodevelopmental disorders is to differentiate patient-specific induced pluripotent stem cells (iPSCs) into neuronal lineages. Besides their applications in regenerative medicine and drug screening (Wu and Hochedlinger, 2011; Trounson and DeWitt, 2016), iPSCs have proved to be a useful tool in multitude of diseases such as Schizophrenia, Alzheimer’s and Parkinson’s disease (Singh et al., 2015). Since its discovery in 2006 the induced pluripotency technology gained an enormous interest and applications were established for a multitude of different research fields (Takahashi and Yamanaka, 2006). The astonishing potential of the iPSC technology is its ability to generate subject-specific cells, which can adopt any lineage of choice (Takahashi et al., 2007). iPSCs differentiation into neuronal cells provides a promising alternative to *ex vivo* human neuronal cells, which cannot be harvested for research purposes for obvious reasons. Moreover, the differentiation process provides a window into human neurodevelopment.

Today, most of the reprogramming approaches are still based on the overexpression of the original factors (i.e., Oct3/4, Klf4, Sox2, and Myc) which delivery mode has been optimized. However, new, more convenient, integration-free systems are immerging, such as RNA vectors or non-integrating viruses (Raab et al., 2014). Originally, iPSCs were generated from fibroblast, but over the years protocols have been developed for the generation of iPSCs from other somatic cells, such as peripheral blood cells and keratinocytes (Aasen and Belmonte, 2010; Staerk et al., 2010). Like fibroblasts, keratinocytes can be harvested from a skin biopsy and used for reprogramming (Ohmine et al., 2012; Piao et al., 2014; Umegaki-Arao et al., 2014; Nakayama et al., 2018), alternatively foreskin samples (Aasen et al., 2008), or plucked hair follicle (Aasen and Belmonte, 2010; Petit et al., 2012). Generating iPSCs from keratinocytes isolated from plucked human hair follicle represents an optimal choice to study neuropsychiatric disorders (protected population under ethical laws), as it circumvents the practice of invasive procedures. Additionally, keratinocytes stem from the same germ layer as neurons (Robicsek et al., 2013), which might mirror in a more reliable way abnormalities in neuronal differentiation, as part of the epigenetic signature may be preserved during reprograming. Serum-free media has the advantage of reducing batch to batch variation, providing more defined standardized conditions that guarantee more reliable downstream experiments (Brunner et al., 2010). Previously described approaches to reprogram follicular keratinocytes are based on the integration of the reprogramming factors into the genome or rely on the use of feeder layers, which might lead to variations in downstream experiments (Aasen and Belmonte, 2010; Novak et al., 2010; Petit et al., 2012; Hung et al., 2015), although growing the keratinocytes on feeder layer might show higher reprogramming efficiency (Aasen et al., 2008; Novak et al., 2010).

Although, several published protocols exist for the iPSCs generation from keratinocytes, we aimed in this study to develop a stable and feasible approach to generate subject-specific neuronal cells by reprogramming follicular keratinocytes. We chose to prioritize reliability by growing the cells in a serum-free and feeder-free environment and using a chemically defined coated surface. The maturation of the generated neuronal cells can be used as a simplified model of human neurodevelopment, where direct manipulation can be performed to identify the involved cellular mechanisms. We aimed to improve the protocol for the generation of integration-free iPSCs from human hair follicle for the study of neurodevelopmental disorder. In the long-term, the described model can be used for example to compare lines derived from ADHD patients and healthy control in order to pinpoint the dysregulated pathways that give rise to a disorder.

## Methods

### Subject Recruitment

Recruitment of three control subjects and one male ADHD patient was performed by a psychiatrist and psychologists of the Department of Child and Adolescent Psychiatry and Psychotherapy, University Hospital of Psychiatry Zurich, University of Zurich. Children and adolescents of both sexes (control subjects: 2/1 male/females) and of Caucasian descent between 8 and 16 years of age (mean = 13.75) were recruited. The diagnosis of ADHD was confirmed according to ICD-10 (World Health Organization, 1992) and DSM-5 (American Psychiatric Association, 2013) criteria. IQ screening was performed using WISC-IV (Wechsler Intelligence test for children; Kaufman et al., 2006) with an exclusion criteria of IQ < 75. To assess comorbidities K-SADS-PL (Kiddie Schedule for Affective Disorders and Schizophrenia, present and lifetime version; Kaufman et al., 1997), was used. The Family Interview for Genetic Studies (parents, FIGS; Nurnberger et al., 1994) was collected to ensure genetic lineage in the subjects. Furthermore, Conners rating scales (Conners, 2001) and CBCL (Child Behavior Checklist) questionnaire (Achenbach and Edelbrock, 1991) were collected. In the case of the ADHD patient, the adherence to drug treatment was assessed by the Medication Adherence Report Scale (MARS-D, German edition; Horne and Hankins, 2004). Exclusion criteria for both healthy controls and the ADHD patient were predominant neurological or psychiatric disorders. Healthy controls without any psychiatric disorder (including no ADHD) were included. Informed written consent was obtained in all cases from the participants and their parents. The Study was approved by the Canton Zurich Ethic Committee (BASEC-Nr.-2016-00101 and BASEC-Nr.-201700825) and followed the latest version of the Declaration of Helsinki.

### Hair Follicle Collection

To pluck the hair follicle, a firm pull motion with forceps was performed at the base of the hair. Only plucked hair in the anagen phase contain enough keratinocytes for a successful culture initiation. Thus, the hairs should ideally have a big visible outer root sheath. Generally, the hairs were plucked from the occipital and temporal scalps regions. The hair shaft was cut slightly behind the follicle with sterile scissors resulting in an approximate 5 mm long piece consisting mainly of the follicle. The plucked hairs were stored in a tube filled with 5 mL Defined keratinocytes-SFM medium (DKSFM; Gibco – Thermo Fisher Scientific, Switzerland) at room temperature and were processed within a few hours after harvesting, although successful keratinocyte culture was achieved with hair follicles plucked up to 1 day before and stored in DKSFM at room temperature. A minimum of five anagen hair follicles was plucked from each subject.

### Isolation and Culture of Keratinocytes

Keratinocyte culture was initiated by following previously described protocols with modifications (Aasen and Belmonte, 2010; Petit et al., 2012). Hair follicles were incubated for 15 min at room temperature in an antibiotic solution containing 1X penicillin and streptomycin (Gibco; i.e., at a final concentration of 100 U/mL penicillin and 100 μg/mL streptomycin). Henceforth, the samples were handled under aseptic conditions. The tissue was dissociated into single cell suspension by a two-step enzymatic digestion with 0.05% Trypsin-EDTA (Gibco) for not more than 10–15 min each round, as keratinocytes are sensitive to this treatment and excessive incubation time might cause loss of cells, followed by inactivation with phosphate-buffered saline (PBS; Gibco) solution containing 10% fetal bovine serum (FBS; Gibco). The cells were centrifuged at 200 ×*g* for 5 min and washed once with 4 mL PBS. After a second centrifugation the pellet was resuspended in DKSFM containing 0.5X penicillin and streptomycin. The cells were seeded on a 24-well plate (Sarstedt, Germany) coated with collagen 1 (“Coating Matrix Kit Protein”; Gibco) and were placed in a humidified incubator at 37°C, 5% CO_2_, and 95% air. After 2 days, the medium was changed to DKSFM without antibiotics and thereafter, the medium was replaced every 2–3 days. Sub-confluent (70–90%) cultures were passaged with 0.05% Trypsin-EDTA and split with a ratio of 1:4 on collagen 1 coated 6-well plate (Sarstedt). Since keratinocytes can lose their proliferating potential after subsequent passaging, low passage (P1-P3) cells were used for further applications.

### Reprogramming of Follicular Keratinocytes

Induced pluripotent stem cells were generated using the CytoTune^TM^-iPS 2.0 kit (Invitrogen – Thermo Fisher Scientific, Switzerland), which is based on three non-integrative Sendai viral vectors (Thermo Fisher) containing polycistronic Klf4-Oct3/4-Sox2 (KOS), cMyc, and Klf4, respectively. Three days before the viral transduction, low passage keratinocytes (Passage 0 or 1) were seeded on collagen 1 coated 6-well plate at a density of 1 × 10^5^ cells/cm^2^, in order to obtain about 60–70% confluency at the time of transduction. A multiplicity of infection (MOI) of 3.2-3.2-2 was used for the three viral vectors, respectively. For each well to be transfected, the calculated volumes of each of the three viruses were added to 1 mL DKSFM supplemented with 5 μM Y-27362 (Adipogen AG, Switzerland), a Rho-associated kinase ROCK inhibitor, prewarmed to 37°C and thoroughly mixed. The medium was removed from the wells and 1 mL of medium containing the viral vectors was added to each well. The cells were placed in a humidified incubator at 37°C 5% CO_2_. The day after, the medium was changed to DKSFM supplemented with 5 μM Y-27362 and in the following days was replaced every other day. Three days after reprogramming, the cells were passaged with TrypLE (Gibco) and placed on a 6-well plate coated with vitronectin (Gibco) substrate at a 1:3 splitting ratio. The medium was progressively switched to Essential 8^TM^ flex (E8, Gibco) with 5 μM Y-27362 8 days after transduction, by increasing the E8 concentration daily to 30, 60, and 100%. From then on, the medium was changed every day, and the cultures were monitored for the emergence of colonies with embryonic stem cell-like morphology. Y-27362 supplementation was discontinued 3 weeks post-transduction. See **Supplementary Figure S1** for an overview of the reprogramming procedure.

### iPSCs Culture

Induced pluripotent stem cells were cultured under serum-free and feeder layer-free conditions using enzyme-free dissociation reagents. Colonies were manually isolated starting from approximately 1 month after reprogramming and fragments were transferred to a vitronectin coated 24 well plate in E8 medium supplemented with 10 μM Y-27362; each colony was placed in a separate well. The next day, the medium was changed with E8 without Y-27362. iPSCs were passaged every 3–4 days using Versene Solution (Gibco) following manufacturer instructions. All the generated iPSC clones were passaged at least 10 times to ensure stabilization of the cultures and to accommodate the cells to the growth conditions. Pluripotency of the cells was assessed using the live-cell staining with TRA-1-60 Alexa Fluor^®^ 488 Conjugate Kit (Invitrogen) following the instructions provided by the manufacturer.

### Neural Differentiation of iPSCs

Induced pluripotent stem cells were induced to adopt neural stem cell (NSC) fate by using “PSC Neural Induction Medium,” which allows for rapid generation of NSC with mid- and forebrain identity from iPSCs cultured in monolayer. Passage 12, 80% confluent, high quality (without visible differentiated cells) iPSC culture was passaged at an estimated cell concentration of 3 × 10^5^ cells per well of a 6-well plate coated with vitronectin. The day after passaging, the medium was aspirated and replaced with 2.5 mL neural induction medium (NIM) composed of Neurobasal^®^ Medium (Gibco) supplemented with 1X Neural Induction Supplement (Gibco) prewarmed at 37°C. After 2 days the medium was replaced with fresh NIM prewarmed at 37°C. At the next medium change (day 4 and 6 of neural induction) the cell reached subconfluency and therefore the medium volume was doubled. After 7 days of culture, the cells reached confluency and were ready for expansion.

Fully confluent NSC cultures were washed once with PBS and the cells were dissociated from the surface using StemPro^®^ Accutase^®^ (Gibco) for 5 min at 37°C. The cells were pelleted by centrifugations for 4 min at 300 ×*g* and resuspended in 4 mL PBS to dilute the dissociation reagent. Again, the cells were pelleted and resuspended in 37°C prewarmed neural expansion medium (NEM) composed of 50% Neurobasal medium, 50% Advanced DMEM/F12 (Gibco) and 1X neural induction supplement. Cells were seeded at 1 × 10^5^ cells/cm^2^ on a Geltrex (Gibco) coated 6-well plate in 2.5 mL NEM supplemented with 5 μM Y-27632. The day after, the medium was replaced with 2.5 mL NEM without Y-27632 and thereafter changed every other day until the culture was ready to be passaged, usually after 4–5 days. For the cryopreservation of NSC, the cells were dissociated using StemPro^®^ Accutase^®^, resuspended in NEM with 10% DMSO and frozen with a “Mr. Frosty” freezing container. Thereafter, the cryo-tubes were transferred into liquid nitrogen tank until use.

Terminal neuronal differentiation was induced by growing NSCs in neural differentiation medium (NDMC) composed of Neurobasal^®^ medium supplemented with 2% B-27^®^(Gibco), 1X Glutamax (Gibco), 200 μM ascorbic acid (Sigma-Aldrich – Merck, Switzerland) and 1X CultureOne^TM^ (Gibco) in double coated Poly-D-Lysine/Laminin (Sigma-Aldrich – Merck) culture vessels. NSCs at passage 4 were dissociated using StemPro^®^ Accutase^®^, the pellet was resuspended in 1 mL NDMC and 5 × 10^4^ cells/cm^2^ were seeded in NDMC supplemented with 5 μM Y-27632. After the cells attached to the surface (usually after 2–3 h), the medium was carefully changed to NDMC without the ROCK inhibitor Y-27632 and the plate was returned to the incubator. The medium was changed every 2–3 days by removing half of the spent medium and adding the same volume of prewarmed NDMC carefully toward the wall of the well, to avoid cell detachment. After 3 weeks of culture, the use of CultureOne^TM^ supplement was discontinued.

### Immunocytochemistry

Cells were fixed with 4% paraformaldehyde (PFA; Santa Cruz Biotechnology, Germany) for 15 min at room temperature. PFA solution was aspirated and the cells were rinsed three times with PBS, 5 min per rinse. Blocking buffer, composed of 0.1% Triton^TM^ X-100 (AppliChem GmbH, Germany) and 1% bovine serum albumin (Sigma-Aldrich – Merck) in PBS, was added for 30 min at room temperature. Rabbit anti-human- β3 Tubulin Class III (TUBB3; BioLegend, United States) was added diluted to a final concentration of 0.5 μg/mL (1:1000) in blocking buffer and incubated at 4°C overnight. The cells were rinsed three times with PBS for 10 min per rinse at room temperature and the secondary antibody anti-rabbit-Alexa-488 (Invitrogen) in blocking buffer was added to the cells at a concentration of 1 μg/mL (1:2000) for 30 min. The wells were rinsed twice with PBS and the slide was mounted with Fluoroshield Mounting Medium with DAPI (Abcam, United Kingdom). Staining was visualized under an IX81microscope (Olympus, Switzerland) with a “DP72” digital camera and the “xcellence RT software” (Olympus, Version 2.0.1). See **Supplementary Material** for antibodies list (**Supplementary Table S1**).

### Quantitative RT-PCR (qRT-PCR)

To extract the RNA, cells were detached, depending on the cell type, with the dissociation reagent of choice and the pellet was resuspended in 1 mL RNAprotect (Qiagen, Germany) and frozen until further use. Total RNA was extracted using RNeasy Plus Mini kit (Qiagen) according to the manufacturer’s instructions. RNA concentration and purity were determined with NanoDrop One spectrophotometer (Thermo Fisher Scientific). One microgram RNA per sample was reverse transcribed into cDNA using iScript^TM^ cDNA Synthesis Kit (Bio-Rad, Switzerland) on the thermocycler CFX96^®^ (Bio-Rad). Gene expression profile was assessed by qRT-PCR using the QuantiTect SYBR Green master mix (Qiagen). Primer sequences were designed with Primer-BLAST (Ye et al., 2012), selected from PrimerBank (Wang and Seed, 2003) or bought as a commercially available primer assay kit. See **Supplementary Material** for the primers details (**Supplementary Table S2**). qRT-PCR was performed using a CFX384^TM^ Real-Time PCR Detection System (Bio-Rad). Each sample was prepared in triplicates, PCR efficiency was calculated using LinRegPCR (Ruijter et al., 2009) and qBasePLUS software (Version 2.3; Biogazelle) was used to determine the normalized mRNA levels. The expression of the examined genes (*KRT14, MAP2, NES, PAX6, SeV, TERT, NANOG, OCT3/4*, and *Lin28A*) was normalized to two or three of the most stably expressed reference genes (*HMBS*, *ACTB*, and *GAPDH*). The expression of the genes used to assess pluripotency was compared to the expression in a commercial hiPSC line (Gibco, catalog number A18945) and isolated RNA from human embryonic stem cell line (HUES6 cell line). To assess the presence of Sendai virus in the generated cell line a primer specific to the viral genome was used. As positive control, SH-SY5Y cells (CRL-226, ATCC) have been transduced with the Sendai virus and RNA was isolated 1 day after. The heatmap plot was generated using the superhot R package.

### Karyotyping

It is well known, that long-term culture induces genetic aberrations in pluripotent cell lines (Liang and Zhang, 2013). Thus, it is important to regularly assess the quality of the genetic material in the different cell lines. Briefly, the cells were treated with 0.1 μg/mL colcemid (Gibco) for 1 h and subsequently collected by trypsinization. Thereafter, the cells were incubated in a hypotonic solution (0.4% KCl) for 25 min at 37°C, fixed with methanol and glacial acetic acid (3:1 ratio) three times and stained with Giemsa stain (Gibco) on a glass slide (Fisher Scientific). The chromosomes were visualized with a 100X objective (Zeiss, Germany) using Ikaros Karyotyping System (MetaSystem, Germany, Version 5.5.5).

### Mycoplasma Contamination Assay

Established cell lines were tested for mycoplasma contamination using the LookOut^®^ Mycoplasma PCR Detection Kit (Sigma-Aldrich – Merck) following the manufacturer’s instructions. PCR was run using a C1000^TM^/CFX96^TM^ Thermal Cycler and the amplified products were loaded onto a 1.2% agarose gel containing HDGreen Plus (INTAS, Germany) diluted 1:20,000 and run at 100 V. Electrophoresis was stopped after 30 min, when the visible bands migrated ca. 2.5 cm and bands were visualized in the “Bio-Rad ChemiDoc^TM^ XRS + System” using “Image Lab^TM^” Software (Bio-Rad, version 5.2.1).

## Results

### Reprogramming of Hair Derived Keratinocytes

**FIGURE 1:**
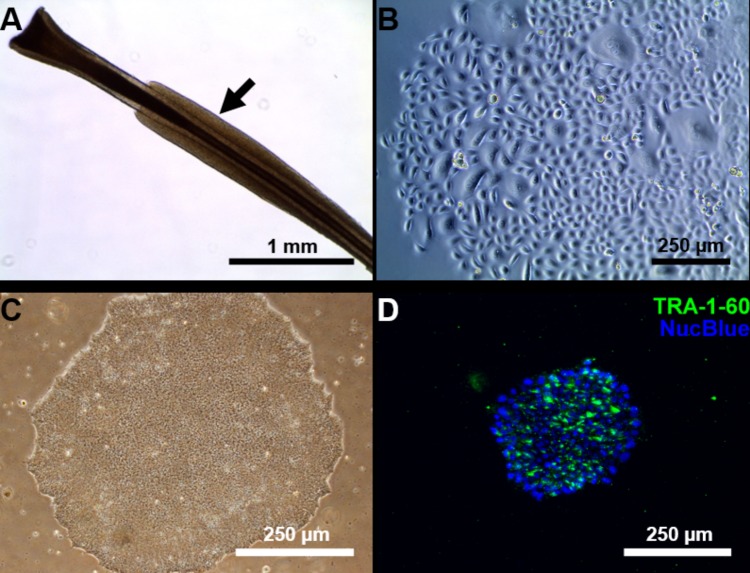
Reprogramming of follicular keratinocytes. **(A)** Plucked human hair follicle from the scalp region. The hair follicle is in the anagen phase. A visible outer root sheath (black arrow) is highly visible and intact, thus providing a higher number of keratinocytes. **(B)** Confluent keratinocyte culture isolated from hair follicles, at 12 days of culture. The cells display typical keratinocyte morphology (strongly adherent cells with cobblestone appearance). **(C)** Representative Sendai viral generated iPSC colonies. Passage 1 iPSC culture manually picked from emerging colony in Cytotune 2.0 transfected keratinocytes. The well-defined borders of the colony are, characteristic of iPSC colonies. **(D)** The generated iPSC culture stains positively for TRA-1-60 (green), a known pluripotency marker. Nucleus is stained with NucBlue (blue).

**FIGURE 2:**
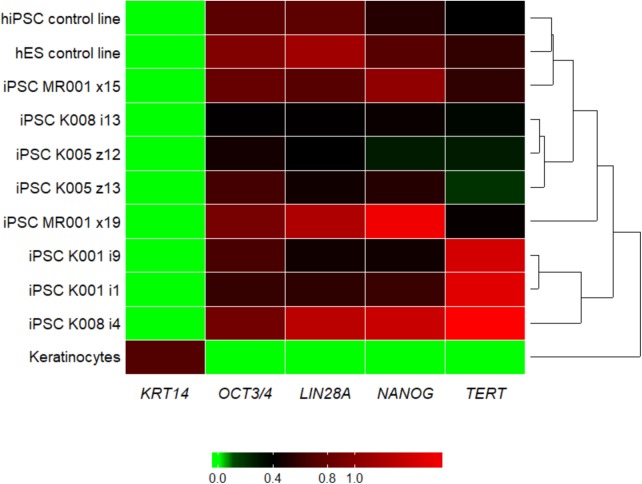
Heatmap showing the qRT-PCR result for the pluripotency markers *LIN28A, NANOG, OCT3/4*, and *TERT* and the keratinocyte marker *KRT14*. Upon reprogramming the keratinocyte marker *KRT14* is highly downregulated, while markers for pluripotency are upregulated in all the generated iPSC lines. For the tested genes, the generated lines show similar expression pattern as the commercial hiPSC line and the embryonic stem cell line. Samples were analyzed in triplicates, normalized to the most stable reference genes (*ACTB, HMBS*, and *GAPDH*). Values were log2 transformed and the plot was generated with the R package superheat (see **Supplementary Table S3**). Levels of high expression are shown with red, while levels of low expression are shown with green. K, control; MR, methylphenidate responder ADHD patient.

We managed to efficiently isolate and culture keratinocytes from a low number of plucked hair follicles in the anagen phase (**Figure 1A**). The isolated cells started to form small colonies of proliferating cells that grew to subconfluency 7–14 days after culture initiation (**Figure 1B**). At this point, keratinocytes were reprogrammed using Sendai viruses containing the reprogramming factors. During the reprogramming phase, ROCK inhibitor was supplemented to reduce post-transduction cell death. At about 3 weeks post-transduction small growing clumps were visible in the culture and the first colonies were usually mechanically isolated 1 week after. For each participant (three healthy controls and one patient with ADHD), two iPSC clones were selected, expanded and used for further experiments. The generated iPSC cultures displayed typical embryonic stem cell morphology (i.e., colony with distinct borders composed of small cells with prominent nuclei; **Figure 1C**). TRA-1-60 staining confirmed the expression of the key pluripotency marker (**Figure 1D**). Furthermore, gene expression analysis revealed an upregulated expression of telomerase reverse transcriptase (*TERT*), *NANOG*, octamer-binding transcription factor 3/4 (*OCT3/4*), lin-28 homolog A (*LIN28A****)*** and a downregulation of the keratinocyte marker keratin 14 (*KRT14*), compared to keratinocyte cultures (**Figure 2** and **Supplementary Table S3** for detailed relative expression). Overall, both the morphology (**Figure 1** and **Supplementary Figure S2**) and the gene expression profiles of the generated iPSC colonies were comparable to the commercial iPSC (**Figure 2**). Moreover, the gene expression of the generated iPSCs was comparable to the RNA from the hESC HUES6 line (**Figure 2**).

**FIGURE 3:**
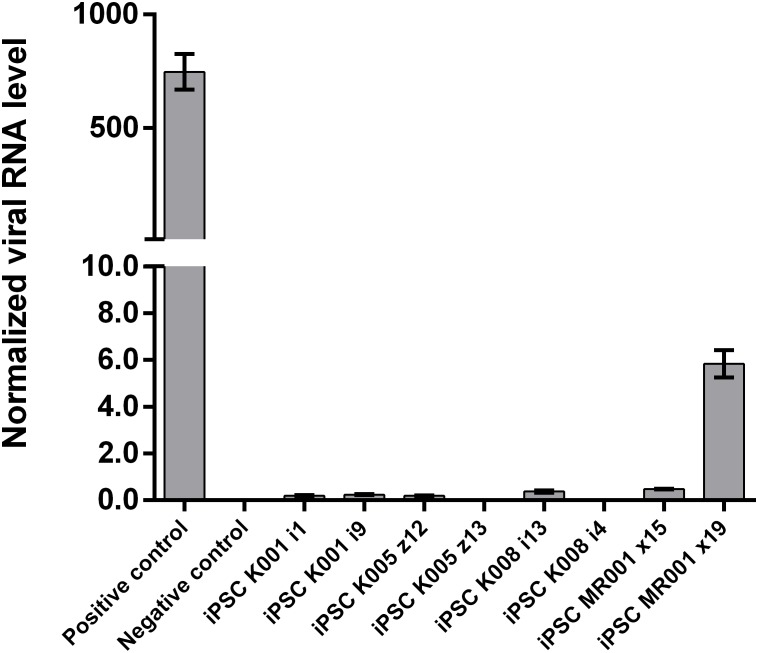
Assessment of Sendai virus traces using qRT-PCR in the cell lines at passage 10. The generated lines show no or little presence of the virus used for reprogramming. Samples were analyzed in triplicates, normalized to the most stable reference genes (ACTB, HMBS, and GAPDH). Values are given as mean ± SEM.

**FIGURE 4:**
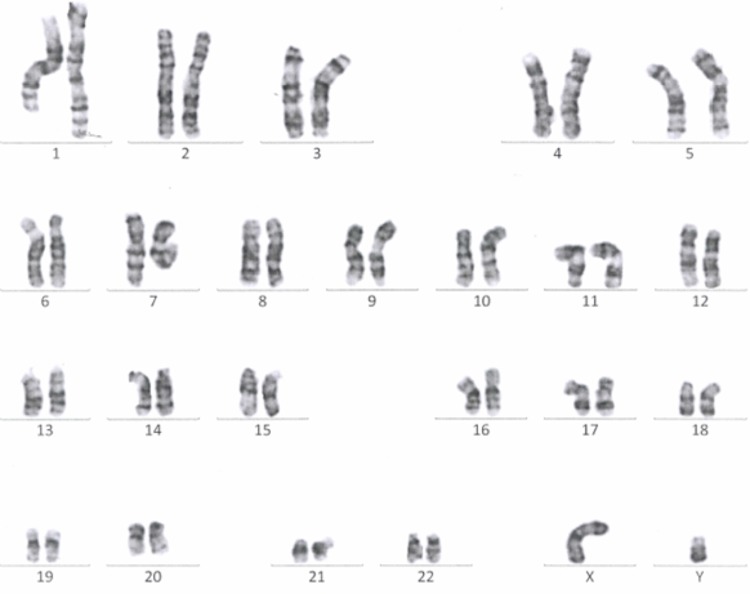
A representative G-banding analysis of a Passage 10 iPSC line. The analyzed clone was karyotypically normal (46, XY), showing no gross genetic aberration.

Passage 10 iPSC cultures were tested for mycoplasma contamination. The absence of a band in the 260 bp range in the electrophoresis gel indicates that the tested samples are not contaminated by mycoplasma (**Supplementary Figure S3**). To assess the absences of the viral vectors used for the reprogramming we performed qRT-PCR in passage 10 iPSC lines using a Sendai specific primer. As a positive control RNA was isolated from SH-SY5Y neuroblastoma cell line transduced with Sendai virus containing hKlf4 at an MOI = 6 three days before harvesting the cells and reversed transcribed to cDNA. For a negative control, untransfected SH-SY5Y cells were used. The analysis showed that one of the eight generated lines were virus-free, while the remaining seven lines showed minimal viral RNA traces (**Figure 3**). G-banding analysis showed that the tested lines have a normal karyotype (**Figure 4**).

### Neuronal Differentiation

**FIGURE 5:**
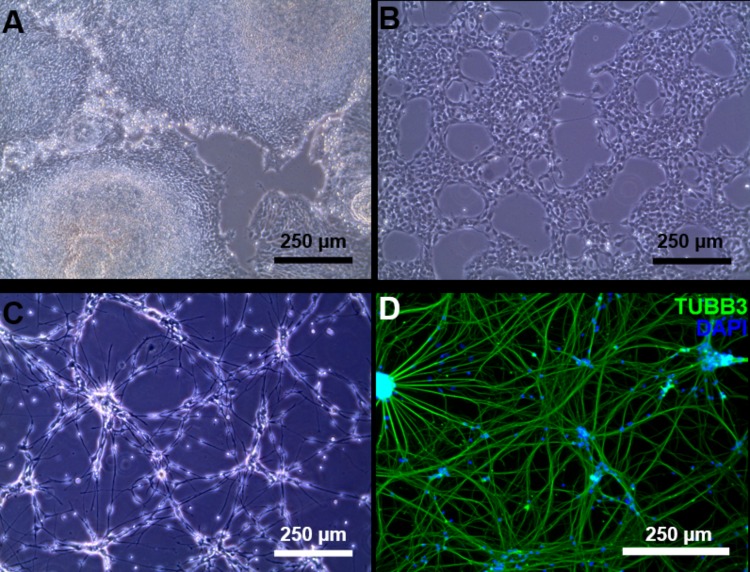
Neuronal differentiation. **(A)** Day 7 of neural induction, the iPSC morphology is lost and the culture is ready to be harvested and expanded. **(B)** Passage 4 NSC culture. **(C)** At day 4 of terminal neuronal differentiation the cells begin to form a neuronal network. **(D)** Tubulin beta 3 class III (green) staining of neuronal cells at day 24 of terminal neuronal differentiation. Counter staining of the nucleus was conducted by DAPI staining (blue).

**FIGURE 6:**
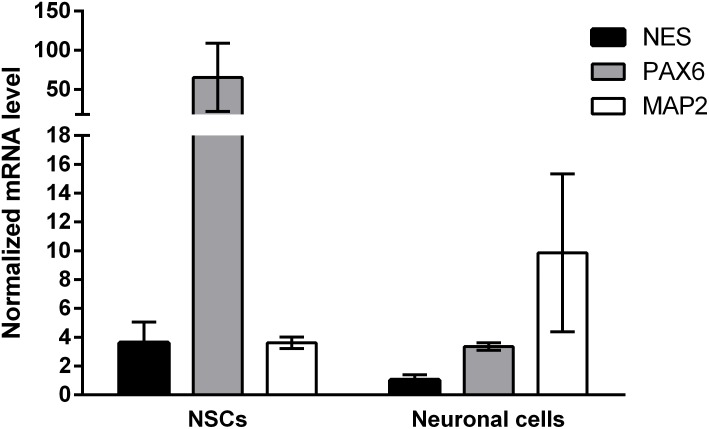
Gene expression results of neuronal differentiation. Samples were assessed for the expression of *PAX6*, *NES*, and *MAP2* and normalized to *ACTB* and *GAPDH* as the most stable reference genes. Neural progenitor makers *PAX6* and *NES* are upregulated in passage 4 NSCs (*N* = 8), while neuronal marker *MAP2* is upregulated in neuronal cells at day 28 of neuronal differentiation (*N* = 2). Values are given as mean ± SEM.

Induced pluripotent stem cells were forced to adapt neural precursor cell fate by using NIM, a commercially available differentiation medium, on Geltrex^®^ coated surface. Within 7 days, the cells showed changes in the morphology, switching from a rather uniform cell distribution in the colony distinctive of iPSC cultures to colonies with tangential polarity (**Figure 5A**). At passage 4 NSCs showed a homogenous morphology (**Figure 5B**) and were differentiated into neuronal cells by plating them on Poly-D-Lysine/Laminin double coated surface and by providing neural induction factors in NDM. Already the day after plating, the cells displayed production of short projections outgrowth from the cell body. On day 4 of differentiation, long neurites were clearly visible (**Figure 5C**). A complex network was formed by day 24 of differentiation and to the cells stained positively for TUBB3, a commonly used neuronal marker (**Figure 5D**). Immunocytochemistry with GFAP antibody showed no positive cells in the culture, indicating that no astrocytes were present (data not shown). Gene expression analysis indicates that NSCs express nestin (*NES*) and paired box 6 (*PAX6*), both markers of neural precursor cells, while During terminal differentiation the cell show expression of microtubule associated protein 2 (*MAP2*, **Figure 6**).

## Discussion

Keratinocytes were successfully isolated and cultured in serum-free media using the described method. We showed that iPSCs can be generated from plucked hair follicles in highly defined cell culture conditions using non-integrative reprogramming vectors. Isolated pluripotent colonies were as well cultured in serum-free media. The described method is an improvement over previously described protocols for the reprogramming of keratinocytes in that we combine using an easily accessible cell source, the use of a non-integrative reprogramming approach and circumvent the use of feeder layers and serum containing medium during the reprogramming (see comparison of published protocols in **Table 1**). We could show that this method is feasible also in generating iPSC from young ADHD patient, though further samples are required to conduct comparison studies with controls at the molecular and cellular levels. Keratinocytes from hair follicle are an excellent choice for sample collection from patients, however, these cells can be rather difficult to maintain *in vitro* when compared to other primary human cells such as fibroblasts by showing less attachment at culture derivation, tendency to differentiate and limited growth potential (Freshney, 2000; Strudwick et al., 2015). We therefore suggest using low passage keratinocytes for the reprogramming experiment. Trypsinization of the hair follicles has proven to be a critical step for the success of the culture initiation. Therefore, short incubation time with low concentrated Trypsin at room temperature was used for the dissociation of the hair follicle and the passaging of keratinocytes. In addition, it is critical that a sufficient number of hair follicles in the anagen phase and with a visible outer root sheath (the tissue layer where the proliferation capable keratinocytes are found) are used. Extensive cell death has been observed when keratinocytes grown in serum/feeder-free conditions are reprogrammed (Aasen and Belmonte, 2010). We found that ROCK inhibitor supplementation improved the post-transduction cell death. It was previously shown that cMyc overexpression induces apoptosis in keratinocytes and the effect can be ameliorated with Y-27632 addition after the transduction (Dakic et al., 2016). The presented method was successful in generating iPSC lines that express pluripotency markers (TRA-1-60, *TERT, NANOG, OCT3/4*, and *LIN28A*) while *KRT14* expression is strongly reduced, similar to the expression profile of embryonic stem cells and the commercial iPSC line. Next, the iPSCs were forced to adopt neuronal lineage using a fast and feasible approach for neuronal differentiation and providing adequate factors. Depending on the application, other neuronal differentiation approaches can be adopted. For example, dopaminergic or glutamatergic differentiation can be induced (Robicsek et al., 2013). If higher complexity is desired, protocols have been described to generate more complex neuronal cultures such as astrocyte-neurons co-cultures (Kuijlaars et al., 2016), or 3D brain organoids (Mariani et al., 2012); these approaches are, however, usually less reproducible and take a longer time to achieve.

**Table 1:** Comparison of current protocol with previous published keratinocyte (KER) reprogramming protocols.

Publication	KER source	KER isolation method	KER growth on feeder-layer (yes/no)	KER cultured in serum containing medium	Reprograming delivery method (yes/no)	Serum containing medium during reprogramming	Reprogramming on feeder-layer (yes/no)	Addition of ROCK inhibitor for reprogramming (yes/no)
Current method	Plucked hairs	Trypsinization	No	No	Sendai Virus	No	No	Yes
Aasen et al., 2008	Foreskin/plucked hair	Follicle outgrowth	No	Yes	Retrovirus	No	Yes	No
Aasen and Belmonte, 2010	Foreskin/skin biopsies/plucked hair	Trypsinization/follicle outgrowth	No	No	Retrovirus	No	Yes	No
Petit et al., 2012	Plucked hair	Trypsinization	Yes	Yes	Lentivirus	No	Yes	No
Mandel et al., 2012	Plucked hair	Trypsinization	Yes	Yes	Lentivirus (excitable)	Yes	Yes	No
Ohmine et al., 2012	Skin biopsy	Enzymatic	No	No	Lentivirus	No	N/a	No
Linta et al., 2013	Plucked hair	Follicle outgrowth	No	No	Lentivirus	No	Yes	No
Umegaki-Arao et al., 2014	Skin biopsy	Enzymatic	N/A	N/A	Retrovirus	No	Yes	No
Piao et al., 2014	Epidermal	Commercial line	No	N/A	Episomal	No	Yes	No
Hung et al., 2015	Plucked hair	Follicle outgrowth	No	No	Episomal	No	Yes	No
Lim et al., 2016	Epidermal	Commercial line	N/A	No	Retrovirus	N/A	Yes	No
Matsumura et al., 2018	Skin biopsy	Enzymatic	Yes	No	Sendai virus	No	Yes	No
Nakayama et al., 2018	Skin biopsy	Enzymatic	Yes	No	Sendai virus	No	Yes	No
Boonkaew et al., 2018	Plucked hair	outgrowth	No	Yes	Sendai virus	No	No	No

*KER, Keratinocyte; N/A, not available.*

## Conclusion

In conclusion, we describe a standardized and relatively simple approach to generate participants-specific neuronal cultures by reprogramming easily accessible somatic cells, namely hair derived keratinocytes. The protocol is intended to be used as a model to study early neurodevelopment by evaluating the differentiation dynamics of the iPSC derived neurons at the molecular level. It is therefore particularly promising for the study of the etiopathology of ADHD and other neurodevelopmental disorders.

## Author Contributions

SR, SW, and EG initiated the conceptual design. SR and EG designed the experimental procedures. SR and AD performed the experiments and the data collection (cell culture, reprogramming, qPCR, and immunocytochemistry). DB-S advised in the initiation of the keratinocytes methodology. AW, SW, and GB initiated the patients’ recruitment design and performed patient and control recruitment. SR drafted and revised the manuscript. All authors reviewed and approved the final manuscript.

## Conflict of Interest Statement

SW has received lecture honoraria from Shire, Opopharma in the last 5 years. Outside professional activities and interests are declared under the link of the University of Zurich http://www.uzh.ch/prof/ssl-dir/interessenbindungen/client/web. The remaining authors declare that the research was conducted in the absence of any commercial or financial relationships that could be construed as a potential conflict of interest.
