# Supplementary material for: Improved Generation of Induced Pluripotent Stem Cells From Hair Derived Keratinocytes – A Tool to Study Neurodevelopmental Disorders as ADHD
Source: Front Cell Neurosci. 2018 Sep 25;12:321. doi: 10.3389/fncel.2018.00321 (PMC6167495; doi:10.3389/fncel.2018.00321)
Supplement: Supplementary file 1 [file Data_Sheet_1.pdf]

1 Supplementary material

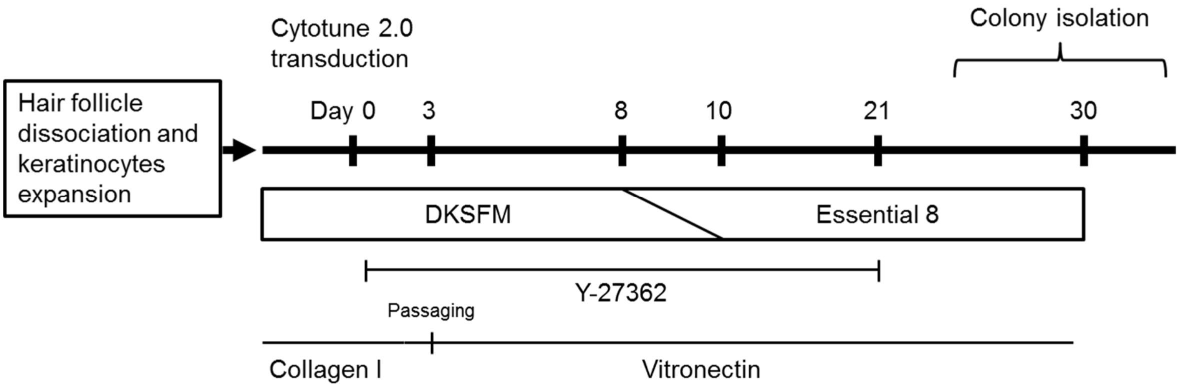

Figure S 1: Overview of the reprogramming of hair derived keratinocytes.

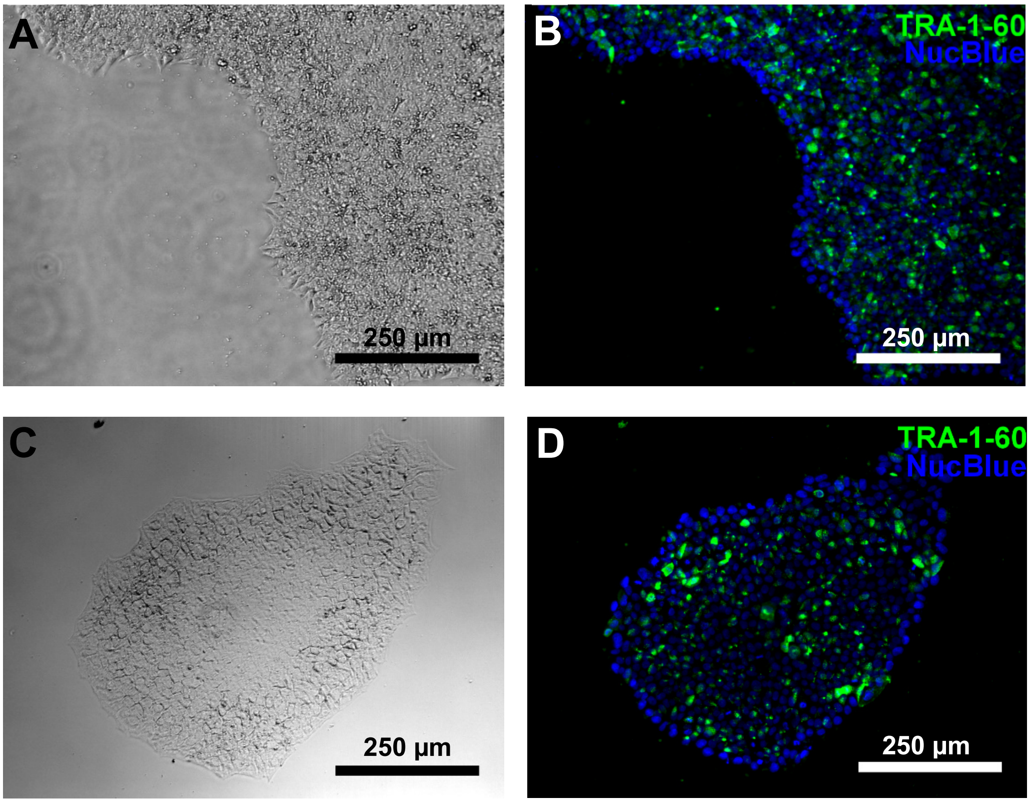

Figure S 2: Comparison between the generated iPSC line and the commercial iPSC line. **(A)** Phase contrast image of a colony from the generated iPSC line. **(B)** TRA-1-60 (green) staining and nucleus staining with NucBlue (blue) shows that generated iPSC line expresses the pluripotency marker. **(C)** Phase contrast image of a colony from the commercial iPSC line. **(D)** TRA-1-60 (green) staining and

nucleus staining with NucBlue (blue) shows that commercial iPSC line expresses the pluripotency marker.

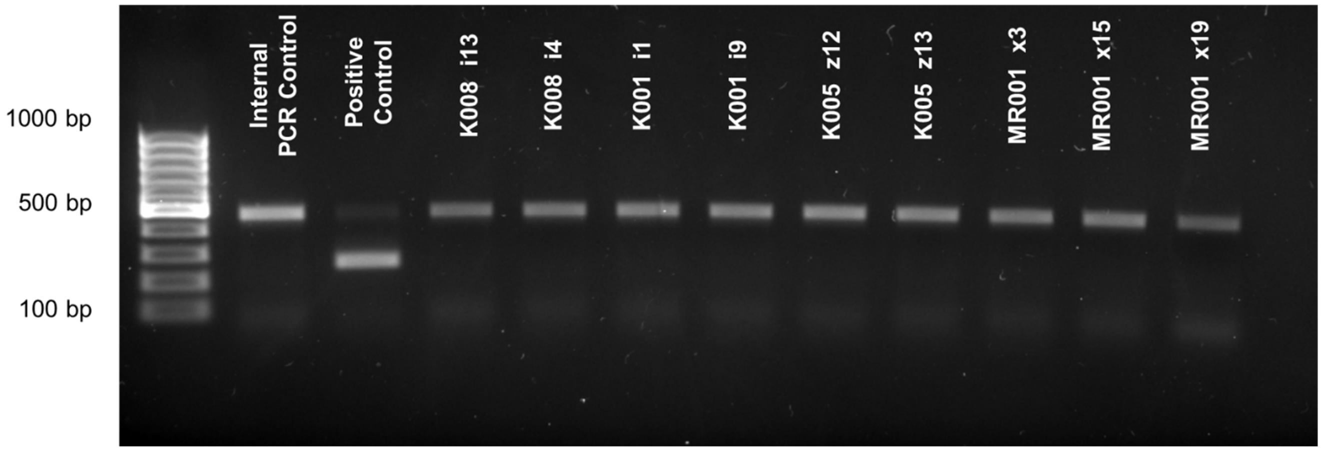

Figure S 3: Mycoplasma contamination assay. PCR products using the mycoplasma detection kit run on a 1.2% agarose gel. Mycoplasma genome in the tested samples was not detected, as indicated by the lack of a ca 260 bp band. The internal control band at ca 480 bp indicates a successfully performed reaction. K, control; MR, methylphenidate responder ADHD patient.

Table S1: Staining material used for the characterization of the neuronal cells.

**Product**	**Manufacturer**	**Cat. No**
Fluoroshield Mounting Medium With DAPI	Abcam	ab104139
Goat anti-Mouse IgG Alexa Fluor 594	Invitrogen	A-11032
Goat anti-Rabbit IgG Alexa Fluor 488	Invitrogen	A-11034
Mouse anti-GFAP	SantaCruz	sc-33673
Rabbit anti-Tubulin β 3 (TUBB3)	BioLegend®	845501

Table S2: List of the primer used for the characterization of the various cell types.

**Gene Symbol**	**Sequence (5’ → 3’)**	**Ann. Temp (°C)**	**Product Length (bp)**	**Meltin g Point Temp (°C)**	**Sequence Source**
TERT	F: TGTCAAGGTGGATGTGACGG<br>R: GAGGAGCTCTGCTCGATGAC	60	267	88.0	Primer-BLAST
NES	F: CAAGATGTCCCTCAGCCTGG<br>R: GGGGTCCTAGGGAATTGCAG	60	144	86.5	Primer-BLAST
LIN28A	F: AGCGCAGATCAAAAGGAGACA<br>R: CCTCTCGAAAGTAGGTTGGCT	60	189	85.5	Primerbank: 94536796c2

PAX6	F: AACGATAACATACCAAGCGTGT<br>R: GGTCTGCCCGTTCAACATC	60	120	81.5	PrimerBank ID 189083679c3
MAP2	F: CGAAGCGCCAATGGATTCC<br>R: TGAACTATCCTTGCAGACACCT	60	161	83.0	PrimerBank ID 87578393c2
KRT14	According to manufacturer	60	76	79.0	Qiagen 249900 (QT00052283)
**Sendai Virus (SeV)**	F: GGATCACTAGGTGATATCGAGC<br>R:ACCAGACAAGAGTTTAAGAGATATGTATC	60	181	81.0	CytoTune 2.0 manual (Invitrogen)
OCT3/4	F: GGA GGA AGC TGA CAA CAA TGA AA<br>R:GGC CTG CAC GAG GGT TT	65	64	78.0	Alonso-Barroso et al., 2017
NANOG	F: TACCTCAGCCTCCAGCAGAT<br>R: CTTCTGCGTCACACCATTGC	58.4	150	82.0	Primer-BLAST
HMBS	According to manufacturer	60	107	83.5	Qiagen 249900 (QT00014462)
ACTB	According to manufacturer	60	146	87.0	Qiagen 249900 (QT00095431)
GAPDH	According to manufacturer	60	95	82.0	Qiagen 249900 (QT00079247)

Table S3: Gene expression values and relative gene expression value (in %) calculated from the generated relative quantities from the pluripotency markers qRT-PCR analysis

	KRT14 (Mean±SEM)	% KRT14 Relative to keratinocyte (Mean±SEM)	LIN28A (Mean±SEM)	% LIN28A Relative to commercial hiPSC line (Mean±SEM)	NANOG (Mean±SEM)	% NANOG Relative to commercial hiPSC line (Mean±SEM)	OCT3/4 (Mean±SEM)	% OCT3/4 Relative to commercial hiPSC line (Mean±SEM)	TERT (Mean±SEM)	% TERT Relative to commercial hiPSC line (Mean±SEM)
Keratinocytes	1.00±0.21	100±29.70	0.00±0	0.00±0	0.00±0	0.00±0	0.00±0	0.00±0	0.00±0	0.00±0
hES control line	0.00±0	0.00±0	2.15±0.13	191.96±18.32	1.05±0.07	147.89±13.25	1.64±0.07	147.75±14.80	0.78±0.10	177.50±28.91
hiPSC commercial line	0.00±0	0.00±0	1.12±0.08	100.00±10.49	0.71±0.04	100.00±8.45	1.11±0.10	100.00±12.87	0.44±0.04	100.00±14.46
iPSC K001 i1	0.00±0	0.00±0	0.76±0.07	67.95±7.80	0.83±0.04	116.90±9.33	0.80±0.08	72.25±9.86	3.57±0.13	811.36±88.08
iPSC K001 i9	0.00±0	0.00±0	0.60±0.03	53.30±4.78	0.59±0.05	83.24±9.26	0.93±0.04	83.96±8.42	3.21±0.31	729.55±88.08
iPSC K005 z12	0.00±0	0.00±0	0.43±0.03	38.75±3.82	0.30±0.02	42.54±4.08	0.61±0.03	54.95±5.74	0.30±0.05	68.64±12.59
iPSC K005 z13	0.00±0	0.00±0	0.59±0.03	52.68±4.79	0.71±0.07	99.72±11.41	0.90±0.08	81.08±10.26	0.20±0.02	46.36±46.36
iPSC K008 i13	0.00±0	0.00±0	0.52±0.04	46.25±4.86	0.56±0.04	79.15±6.98	0.52±0.04	46.49±5.84	0.40±0.05	90.45±15.01
iPSC K008 i4	0.00±0	0.00±0	2.68±0.31	239.29±32.88	2.98±0.35	419.72±55.18	1.41±0.19	127.03±20.88	25.20±3.19	5727.27±932.05
iPSC MR001 x15	0.00±0	0.00±0	1.06±0.05	94.64±8.37	1.88±0.16	264.79±27.76	1.25±0.06	112.61±11.56	0.77±0.11	174.55±31.28
iPSC MR001 x19	0.00±0	0.00±0	2.44±0.10	217.86±18.32	4.06±0.42	571.83±68.43	1.48±0.06	133.33±13.32	0.54±0.09	121.82±23.18
